# Association of preoperative controlling nutritional status score with clinical outcomes among surgical patients with esophageal cancer: a meta-analysis

**DOI:** 10.3389/fonc.2025.1694236

**Published:** 2025-11-11

**Authors:** Yanli Ji, Lei Wang, Xiaoli Mei, E Zheng, Lin Lin, Mei Yang

**Affiliations:** Department of Thoracic Surgery, West China Hospital, Sichuan University/West China School of Nursing, Sichuan University, Chengdu, China

**Keywords:** controlling nutritional status score, esophageal cancer, surgery, long-term outcomes, short-term outcomes, meta-analysis

## Abstract

**Purpose:**

The aim of this study was to identify the relationship between preoperative controlling nutritional status (CONUT) score and long-term and short-term outcomes in patients with esophageal cancer receiving esophagectomy.

**Methods:**

The Web of Science, EMBASE, PubMed, and CNKI databases were searched up to 24 January 2025. Primary outcome was long-term survival such as the overall survival (OS), disease-free survival (DFS), and cancer-specific survival (CSS). Secondary outcomes included the postoperative overall complication, incision infection, anastomotic fistula, pneumonia, respiratory complication, 90-day death, cardiovascular complication, major adverse cerebrocardiovascular event (MACCE), pulmonary atelectasis, and pulmonary embolism. Hazard ratio (HR) and odds ratio (OR) with 95% confidence interval (CI) were separately combined for the primary and secondary outcomes. Subgroup analysis for the OS and DFS by neoadjuvant therapy and pathological type was further conducted.

**Results:**

Eighteen studies with 5,495 cases were included. Pooled results manifested that elevated preoperative CONUT score predicted significantly worse OS (HR = 1.75, 95% CI: 1.30–2.37, *p*<0.001), DFS (HR=1.21, 95% CI: 1.13–1.30, *p* < 0.001), and CSS (HR = 2.60, 95% CI: 1.65–4.10, *p*<0.001). Subgroup analysis for the OS and DFS by the history of neoadjuvant therapy and pathological type demonstrated similar results. Furthermore, elevated CONUT score was significantly related to increased risk of overall complication (OR = 1.50, 95% CI: 1.14–1.96, *p*=0.004), pneumonia (OR= 1.60, 95% CI: 1.23–2.08, *p*<0.001), respiratory complication (OR = 1.60, 95% CI: 1.26–2.03, *p*<0.001), cardiovascular complication (OR = 3.660, 95% CI: 1.068–12.550, *p*=0.039), MACCE (OR = 1.920, 95% CI: 1.068–3.452, *p*=0.040), and pulmonary atelectasis (OR = 2.314, 95% CI: 1.408–3.805, *p*<0.001).

**Conclusion:**

Preoperative CONUT score might serve as a prognostic indicator in surgical esophageal cancer, and patients with elevated CONUT score are suggested to experience worse long-term and short-term clinical outcomes.

## Introduction

Over the past few decades, remarkable advances in cancer treatment—including surgery, chemotherapy, radiotherapy, immunotherapy, and targeted therapy—have substantially improved patient survival and quality of life (1). Nevertheless, despite these therapeutic breakthroughs, certain malignancies, such as esophageal cancer, remain associated with high incidence and mortality rates, particularly in regions such as China (2). According to the World Health Organization, the global incidence of esophageal cancer has shown an upward trend, especially among male patients (3, 4). Major risk factors include long-term smoking, alcohol consumption, poor dietary habits, and chronic esophageal conditions such as esophagitis and gastroesophageal reflux (5, 6). Surgical treatment continues to serve as the cornerstone for early-stage or localized esophageal cancer, while multimodal therapies combining surgery with chemotherapy and radiotherapy are commonly applied for locally advanced disease (5). However, because of the biological heterogeneity of esophageal cancer, treatment outcomes after surgery vary widely among patients, underscoring the importance of accurate prognostic assessment to guide postoperative management.

Postoperative prognosis prediction for esophageal cancer is a significant challenge. In the short term, factors such as surgical complications, postoperative recovery, and comorbidities may impact patient recovery. Long-term prognosis, on the other hand, is influenced by multiple factors, including tumor staging, lymph node metastasis, and the patient’s overall health status. While certain prognostic tools (such as TNM staging and tumor marker detection) are available, the existing methods still have limitations due to the heterogeneity of esophageal cancer and individual patient differences (7, 8). Consequently, identifying more accurate short-term and long-term prognostic indicators has become a key focus in current esophageal cancer research.

Controlling nutritional status (CONUT) score is a simple, cost-effective, and reliable nutritional assessment tool, derived from indicators such as serum albumin, total cholesterol levels, and peripheral blood lymphocyte count. In patients with cancer, particularly those undergoing surgery, chemotherapy, or radiotherapy, there is a high risk of malnutrition, which can impair immune function, postoperative recovery, and treatment tolerance, ultimately affecting treatment outcomes and survival rates (9, 10). Research has shown that patients with a higher CONUT score generally have poorer nutritional status, suppressed immune function, and worse cancer prognosis (11). Conversely, patients with a lower CONUT score typically have better nutritional status, faster postoperative recovery, and longer survival. The prognostic value of CONUT has been well-established in various types of cancer such as gastric and colorectal cancer (11, 12). However, it remains unclear whether CONUT can effectively predict the short-term and long-term prognosis of patients with esophageal cancer undergoing surgical treatment.

This study aimed to further identify the relationship between preoperative CONUT score and long-term and short-term outcomes in patients with esophageal cancer receiving esophagectomy.

## Materials and methods

The current meta-analysis was performed according to the Preferred Reporting Items for Systematic Reviews and Meta-Analyses 2020 (13).

### Literature search

The Web of Science, EMBASE, PubMed, and CNKI databases were searched from database inception up to 24 January 2025 with the following terms: controlling nutritional status core, CONUT, esophageal, esophagus, tumor, cancer, neoplasm, and carcinoma. Detailed search strategy in the PubMed was as follows: (controlling nutritional status core OR CONUT) AND (esophageal OR esophagus) AND (tumor OR cancer OR neoplasm OR carcinoma) (**Supplementary File 1**). MeSH terms and free texts were applied. Moreover, all references in included studies were also reviewed.

### Inclusion criteria

Studies that met the following criteria were included: (1) patients were diagnosed with primary esophageal cancer and receiving the surgical therapy; (2) the CONUT score was evaluated before the surgery based on the cholesterol level, serum albumin level, and total lymphocyte level according to the previous published formula (14); (3) the association of preoperative CONUT score with at least one of the clinical outcomes was explored; (4) articles were published in English or Chinese; and (5) full texts were available.

### Exclusion criteria

Studies that met the following criteria were excluded: (1) letters, editorials, case reports, reviews, or animal trials; and (2) duplicate or overlapping data.

### Data collection

The following information was collected: the name of the first author, publication year, country, sample size, history of neoadjuvant therapy, pathological type, comparison of CONUT score, endpoint, hazard ratio (HR), odds ratio (OR), and 95% confidence interval (CI).

Primary outcome was long-term survival including the overall survival (OS), disease-free survival (DFS), and cancer-specific survival (CSS). Secondary outcomes were postoperative complications including the overall complication, incision infection, anastomotic fistula, pneumonia, respiratory complication, 90-day death, cardiovascular complication, major adverse cerebrocardiovascular event (MACCE), pulmonary atelectasis, and pulmonary embolism.

### Methodological quality assessment

The quality of included studies was assessed by the Newcastle–Ottawa Scale (NOS) score tool and studies with a NOS score >5 were defined as high-quality studies (15).

Two authors independently performed the literature search, study selection, data extraction, and methodological quality assessment. The inter-rater reliability between the two reviewers was assessed, and any discrepancies were resolved through discussion and consensus with a third investigator.

### Statistical analysis

Statistical analyses were performed by STATA (version 15.0) software. Heterogeneity between included studies was assessed by *I*^2^ statistics. If significant heterogeneity was detected (*I*^2^ > 50%), the random-effects model was applied; otherwise, the fixed-effects model was applied. HRs and 95% CIs were combined to evaluate the association between CONUT score and survival. ORs and 95% CIs were combined to evaluate the predictive role of postoperative complications. Sensitivity analysis was conducted to detect the sources of heterogeneity and assess the stability of the overall results. Meanwhile, Begg’s funnel plot and Egger’s test were conducted to detect publication bias (16, 17).

## Results

### Literature search and selection

**Figure 1:**
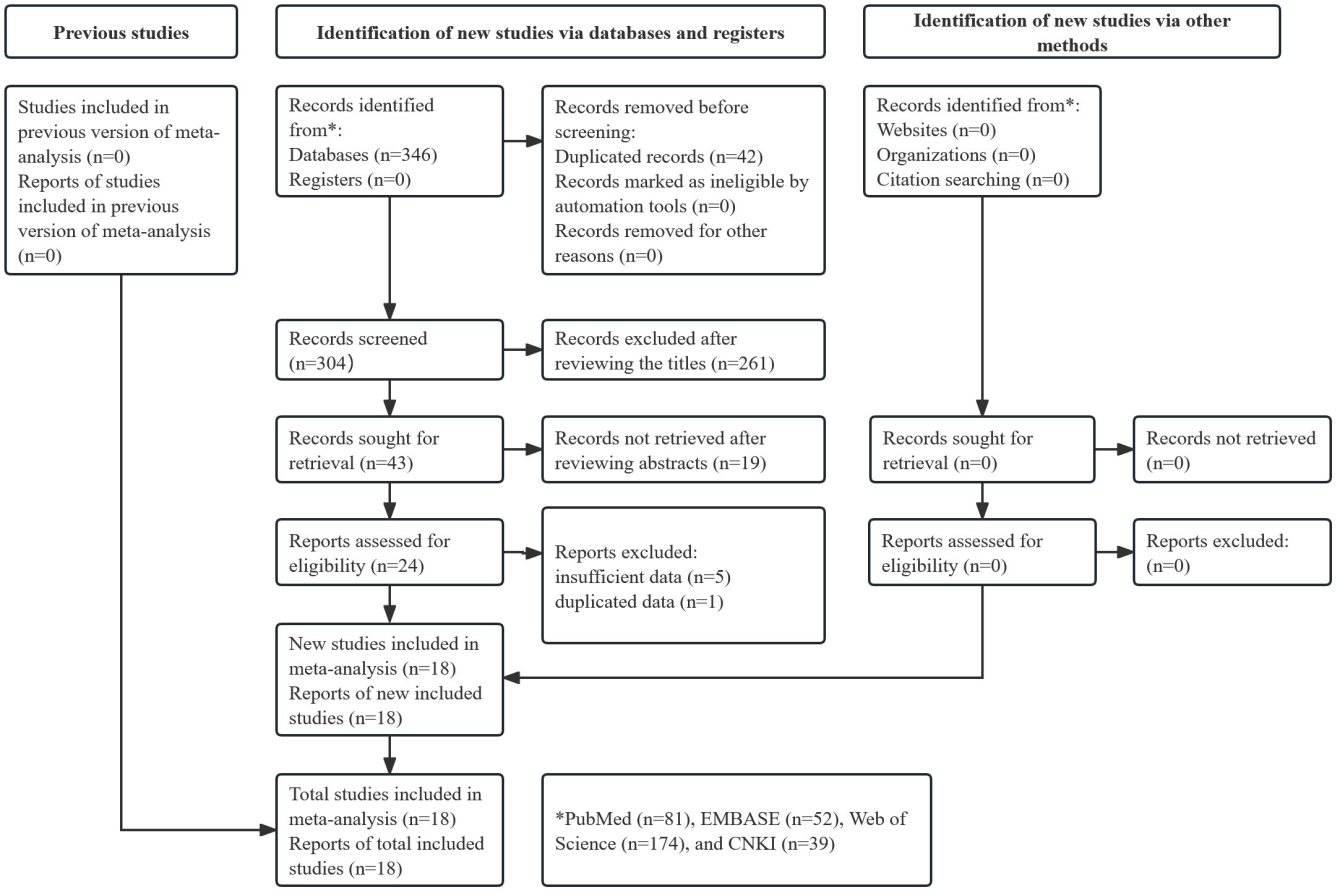
Flow diagram of this meta-analysis.

**Figure 1** presents the literature search and selection process. Initially, 346 records were searched from databases and 42 duplicated records were removed. After reviewing titles, abstracts, and full texts, 286 publications were excluded. A total of 18 studies were included in this meta-analysis (18–35).

### Basic characteristics of included studies

Among the 18 included studies, 5,495 patients were involved. Most studies were from China or Japan and focused on patients with squamous cell carcinoma (SCC). The sample size ranged from 67 to 1,265. The NOS scores of all included studies were all higher than 5, with detailed information in **Supplementary Table 1**. Other specific data are shown in **Table 1**.

**Table 1:** Basic characteristics of included studies.

Author	Year	Country	Sample size	Neoadjuvant therapy	Pathological type	Comparison of CONUT score	Endpoint	NOS
Toyokawa (18)	2016	Japan	185	Mixed	SCC	0–2 vs. 3–12	OS, DFS	7
Yoshida (19)	2017	Japan	373	Mixed	NR	0–4 vs. 5–12	OS, CSS	7
Hirahara (20)	2018	Japan	148	No	SCC	0–1 vs. 2–12	CSS	7
Xu (21)	2018	China	510	No	SCC	0–1 vs. 2–4 vs. 5–8 vs. 9–12	OS, complication	8
Hikage (22)	2019	Japan	141	Chemotherapy	NR	0–4 vs. 5–12	OS, DFS	7
Sakai (23)	2020	Japan	105	No	NR	0–2 vs. 3–12	OS	6
Yoon (24)	2021	Korea	1265	NR	SCC	0–2 vs. 3–12	OS, DFS, 90-day death, MACCE, II, RC	8
Urabe (25)	2021	Japan	224	Mixed	Mixed	0–1 vs. 2–4 vs. 5–12	OS, CSS, complication	7
Wang (26)	2021	China	192	Mixed	SCC	0–3 vs. 4–12	Complication	7
Feng (27)	2022	China	216	Immunochemotherapy	SCC	0–2 vs. 3–12	DFS	7
He (28)	2022	China	214	No	Mixed	0–1 vs. 2–12	Complication, pneumonia, PE, AF, PA, II	7
Horinouchi (29)	2022	Japan	674	Mixed	NR	0–4 vs. 5–12	Complication, RC, II, AF, CVC	8
Fujiwara (30)	2023	Japan	118	Mixed	SCC	0–1 vs. 2–12	OS, DFS	7
Nonogaki (31)	2023	Japan	464	Mixed	SCC	0–1 vs. 2–4 vs. 5–12	OS, complication, pneumonia,	7
Yu (32)	2023	China	67	No	SCC	0–1 vs. 2–4 vs. 5–8 vs. 9–12	OS	6
Fang (33)	2024	China	314	Yes (mixed)	SCC	0–4 vs. 5–12	OS, DFS, complication	7
Gao (34)	2024	China	100	Chemotherapy	Mixed	<3.05 vs. ≥3.05	OS	7
Kubo (35)	2024	Japan	185	Chemotherapy	Mixed	0–4 vs. 5–12	DFS	7

NR, not reported; SCC, squamous cell carcinoma; PE, pulmonary embolism; AF, anastomotic fistula; PA, pulmonary atelectasis; II, incision infection; RC, respiratory morbidity; CVC, cardiovascular complication; MACCE, major adverse cerebrocardiovascular events; OS, overall survival; DFS, disease-free survival; CSS, cancer-specific survival; NOS, Newcastle–Ottawa Scale.

### Association between preoperative CONUT score and primary outcomes

**Figure 2:**
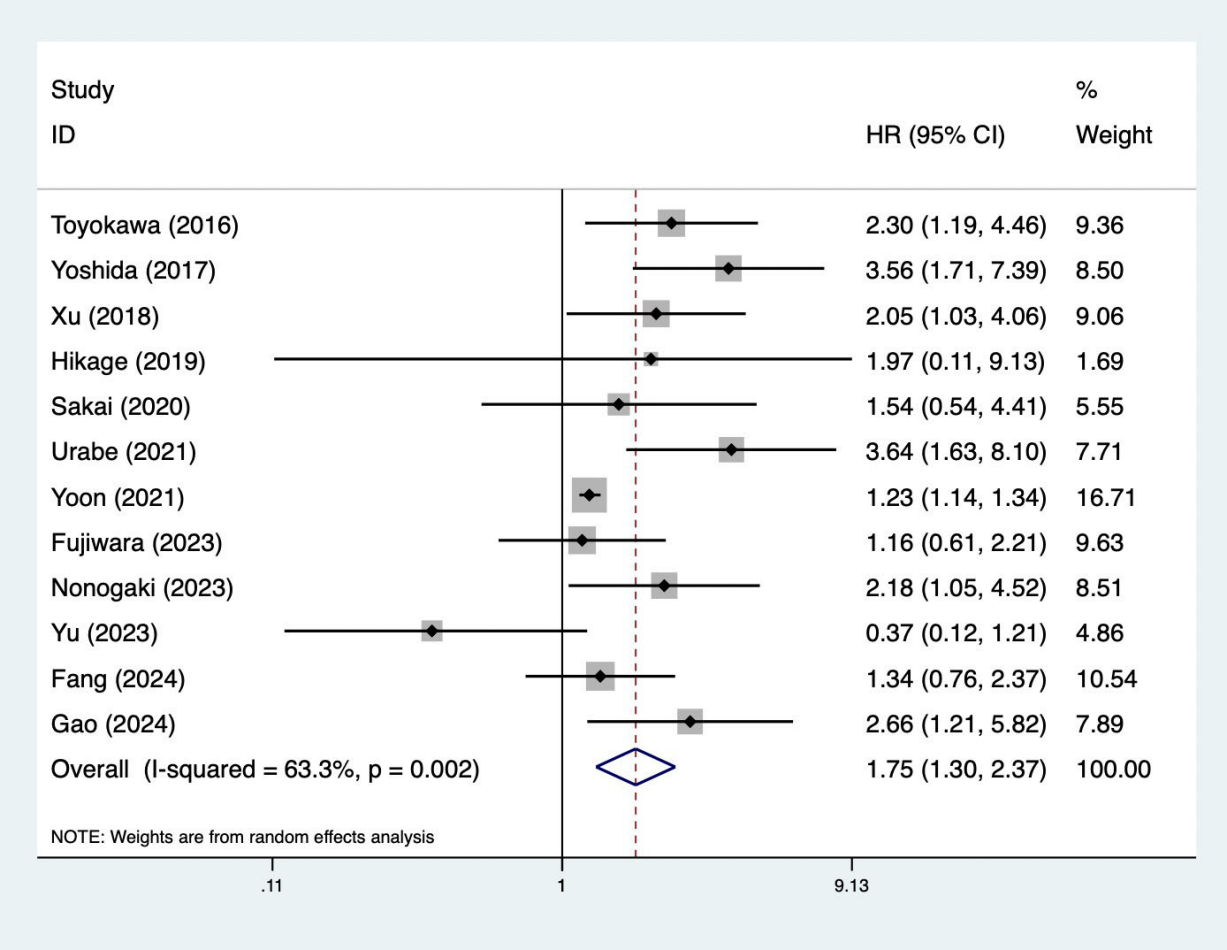
Association of preoperative controlling nutritional status score with overall survival among surgical patients with esophageal cancer.

Fifteen studies explored the predictive role of preoperative CONUT score for long-term survival among surgical patients with esophageal cancer (18–25, 27, 30–35). Pooled results demonstrated that elevated preoperative CONUT score was related to decreased OS (HR = 1.75, 95% CI: 1.30–2.37, *p*<0.001; *I*^2^ = 63.3%, *p*=0.002) (**Figure 2**). Subgroup analysis based on the history of neoadjuvant therapy (mixed: HR = 2.30, 95% CI: 1.51–3.50, *p*<0.001; *I*^2^=42.9%, *p*=0.136; no: HR = 1.14, 95% CI: 0.43–3.02, *p*=0.794; *I*^2^ = 68.3%, *p*=0.043; yes: HR = 1.71, 95% CI: 1.09–2.68, *p*=0.020; *I*^2^ = 0.0%, *p*=0.380) (**Supplementary Figure 1A**) manifested similar results, although the association between CONUT score and OS among patients without history of neoadjuvant therapy did not reach the statistical difference. Subgroup analysis by the pathological type (SCC: HR = 1.41, 95% CI: 1.06–1.89, *p*= 0.019; *I*^2^ = 50.2%, *p*=0.061; mixed type: HR = 3.10, 95% CI: 1.77–5.43, *p*<0.001; *I*^2^ = 0.0%, *p*=0.582) (**Supplementary Figure 1B**) also indicated the association between CONUT score and OS (**Table 2**).

**Table 2:** Results of meta-analysis for primary outcomes.

Items	Number of studies	Hazard ratio	95% confidence interval	*p*-value	*I*^2^ (%)	*p*-value
Overall survival	12	1.75	1.30–2.37	<0.001	63.3	0.002
History of neoadjuvant therapy						
Mixed	5	2.30	1.51–3.50	<0.001	42.9	0.136
No	3	1.14	0.43–3.02	0.794	68.3	0.043
Yes	3	1.71	1.09–2.68	0.020	0.0	0.380
Pathological type						
Squamous cell carcinoma	7	1.41	1.06–1.89	0.019	50.2	0.061
Mixed pathological type	2	3.10	1.77–5.43	<0.001	0.0	0.582
Disease-free survival	8	1.21	1.13–1.30	<0.001	34.3	0.154
History of neoadjuvant therapy						
Mixed	3	1.82	1.23–2.70	0.003	21.4	0.280
Yes	4	1.64	1.13–2.39	0.010	0.0	0.818
Pathological type						
Squamous cell carcinoma	5	1.20	1.12–1.30	<0.001	41.7	0.143
Mixed pathological type	2	2.36	1.13–4.92	0.022	0.0	0.439
Cancer-specific survival	2	2.60	1.65–4.10	<0.001	25.7	0.246

**Figure 3:**
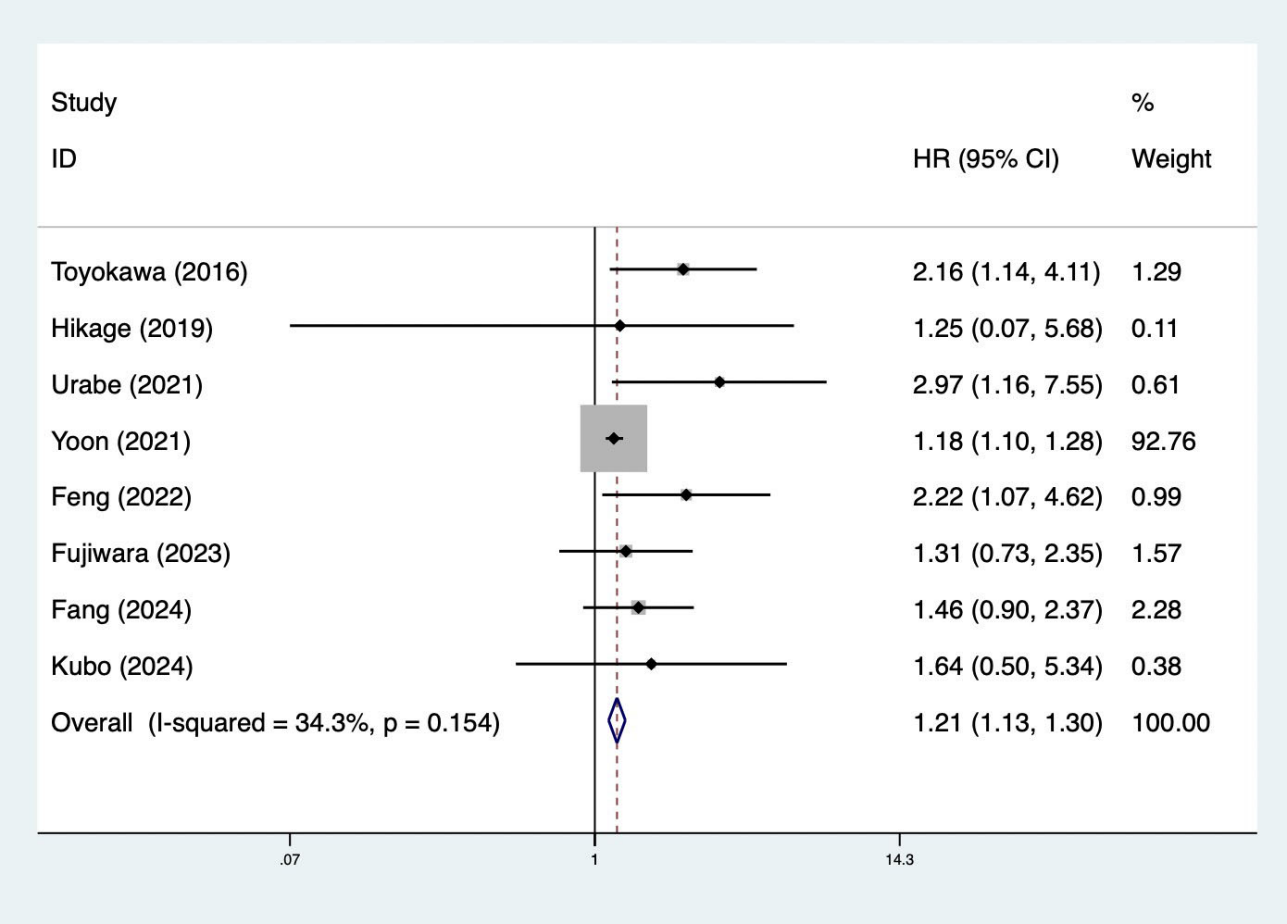
Association of preoperative controlling nutritional status score with disease-free survival among surgical patients with esophageal cancer.

Furthermore, preoperative CONUT score was associated with DFS (HR = 1.21, 95% CI: 1.13–1.30, *p*<0.001; *I*^2^ = 34.3%, *p*=0.154) (**Figure 3**). Meanwhile, subgroup analysis stratified by the history of neoadjuvant therapy (mixed: HR = 1.82, 95% CI: 1.23–2.70, *p*=0.003; *I*^2^ = 21.4%, *p*=0.260; yes: HR = 1.64, 95% CI: 1.13–2.39, *p*=0.010; *I*^2^ = 0.0%, *p*=0.818) (**Supplementary Figure 1C**) and pathological type (SCC: HR = 1.20, 95% CI: 1.12–1.30, *p*<0.001; *I*^2^ = 41.7%, *p*=0.143; mixed type: HR = 2.36, 95% CI: 1.13–4.92, *p*=0.022; *I*^2^ = 0.0%, *p*=0.439) (**Supplementary Figure 1D**) indicated consistent results (**Table 2**).

Furthermore, elevated preoperative CONUT score predicted worse CSS (HR = 2.60, 95% CI: 1.65–4.10, *p*<0.001; *I*^2^ = 25.7%, *p*=0.246) (**Table 2**).

### Association between preoperative CONUT score and secondary outcomes

**Figure 4:**
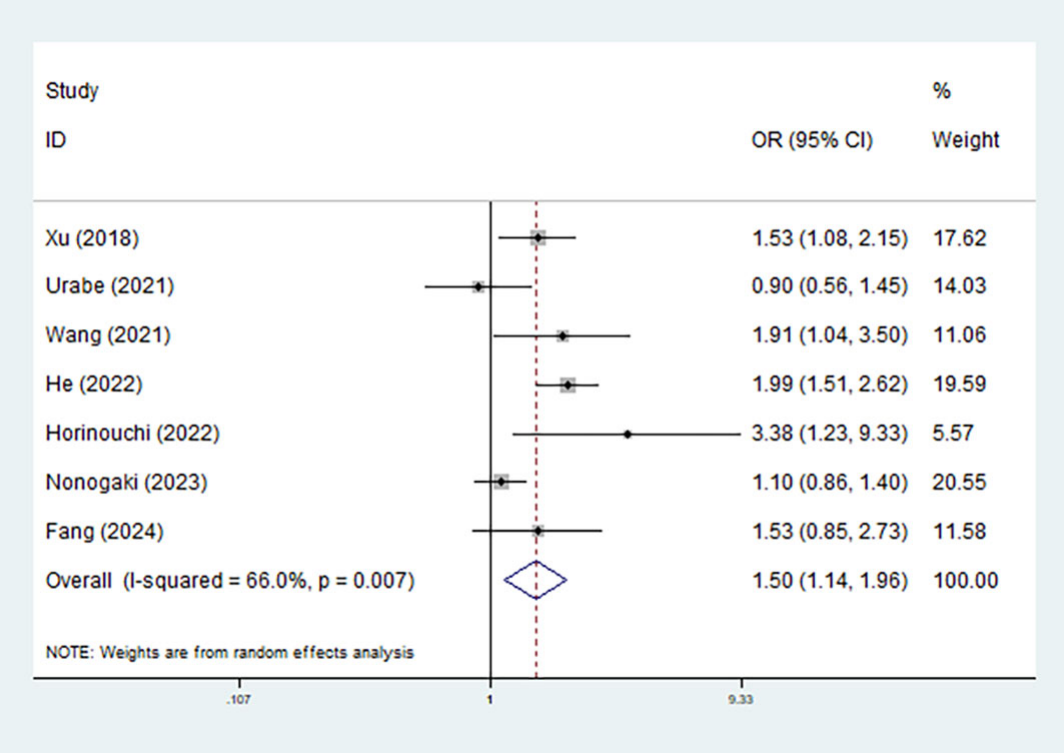
Association of preoperative controlling nutritional status score with postoperative overall complications among surgical patients with esophageal cancer.

Eight studies explored the relationship between the CONUT score and the risk of postoperative complications (21, 24–26, 28, 29, 31, 33). Overall, pooled results demonstrated that elevated preoperative CONUT score predicted increased risk of postoperative complications (OR = 1.50, 95% CI: 1.14–1.96, *p*=0.004; *I*^2^ = 66.0%, *p* =0.007) (**Figure 4**). For specific complications, elevated CONUT score was significantly related to increased risk of pneumonia (OR = 1.60, 95% CI: 1.23–2.08, *p* <0.001; *I*^2^ = 25.6%, *p*=0.246) (**Supplementary Figure 2A**), respiratory complication (OR = 1.60, 95% CI: 1.26–2.03, *p*<0.001; *I*^2^ = 44.8%, *p*=0.178) (**Supplementary Figure 2B**), cardiovascular complication (OR = 3.660, 95% CI: 1.068–12.550, *p*=0.039), MACCE (OR = 1.920, 95% CI: 1.068–3.452, *p*=0.040), and pulmonary atelectasis (OR = 2.314, 95% CI: 1.408–3.805, *p*<0.001) (**Table 3**).

**Table 3:** Results of meta-analysis for secondary outcomes.

Items	Number of studies	Odds ratio	95% confidence interval	*p*-value	*I*^2^ (%)	*p*-value
Overall complication	7	1.50	1.14–1.96	0.004	66.0	0.007
Incision infection	3	0.98	0.65–1.47	0.923	47.4	0.149
Anastomotic fistula	2	1.73	0.91–3.27	0.095	0.0	0.677
Pneumonia	2	1.60	1.23–2.08	<0.001	25.6	0.246
Respiratory complication	2	1.60	1.26–2.03	<0.001	44.8	0.178
90-day death	1	2.012	1.095–3.698	0.034	–	–
Cardiovascular complication	1	3.660	1.068–12.550	0.039	–	–
Major adverse cerebrocardiovascular events	1	1.920	1.068–3.452	0.040	–	–
Pulmonary atelectasis	1	2.314	1.408–3.805	<0.001	–	–
Pulmonary embolism	1	2.030	0.576–7.159	0.402	–	–

### Sensitivity analysis and publication bias

**Figure 5:**
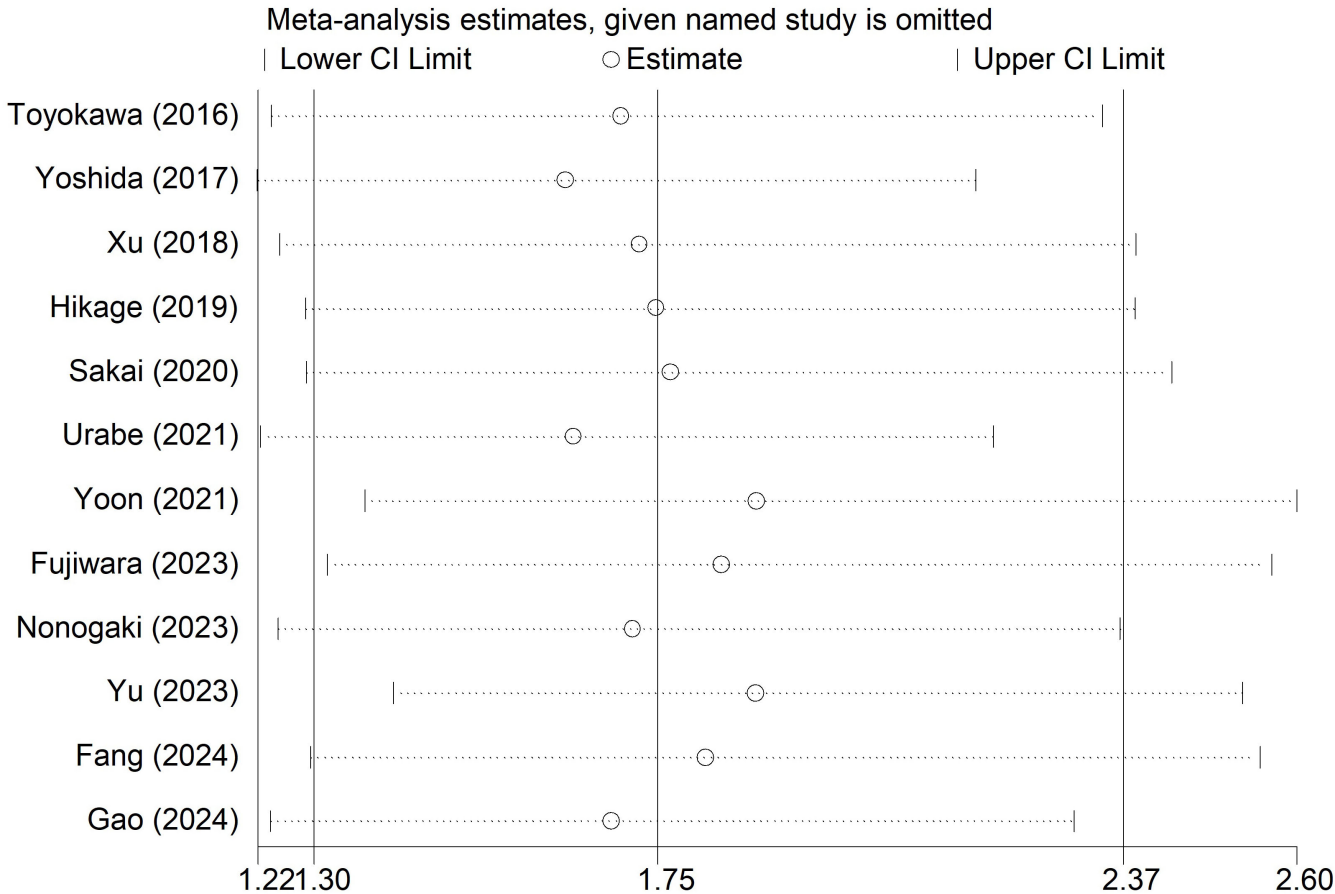
Sensitivity analysis for the association of preoperative controlling nutritional status score with overall survival among surgical patients with esophageal cancer.

Sensitivity analysis for the OS was conducted (**Figure 5**), which manifested that our results were stable and none of the included studies caused an obvious impact on the overall findings.

**Figure 6:**
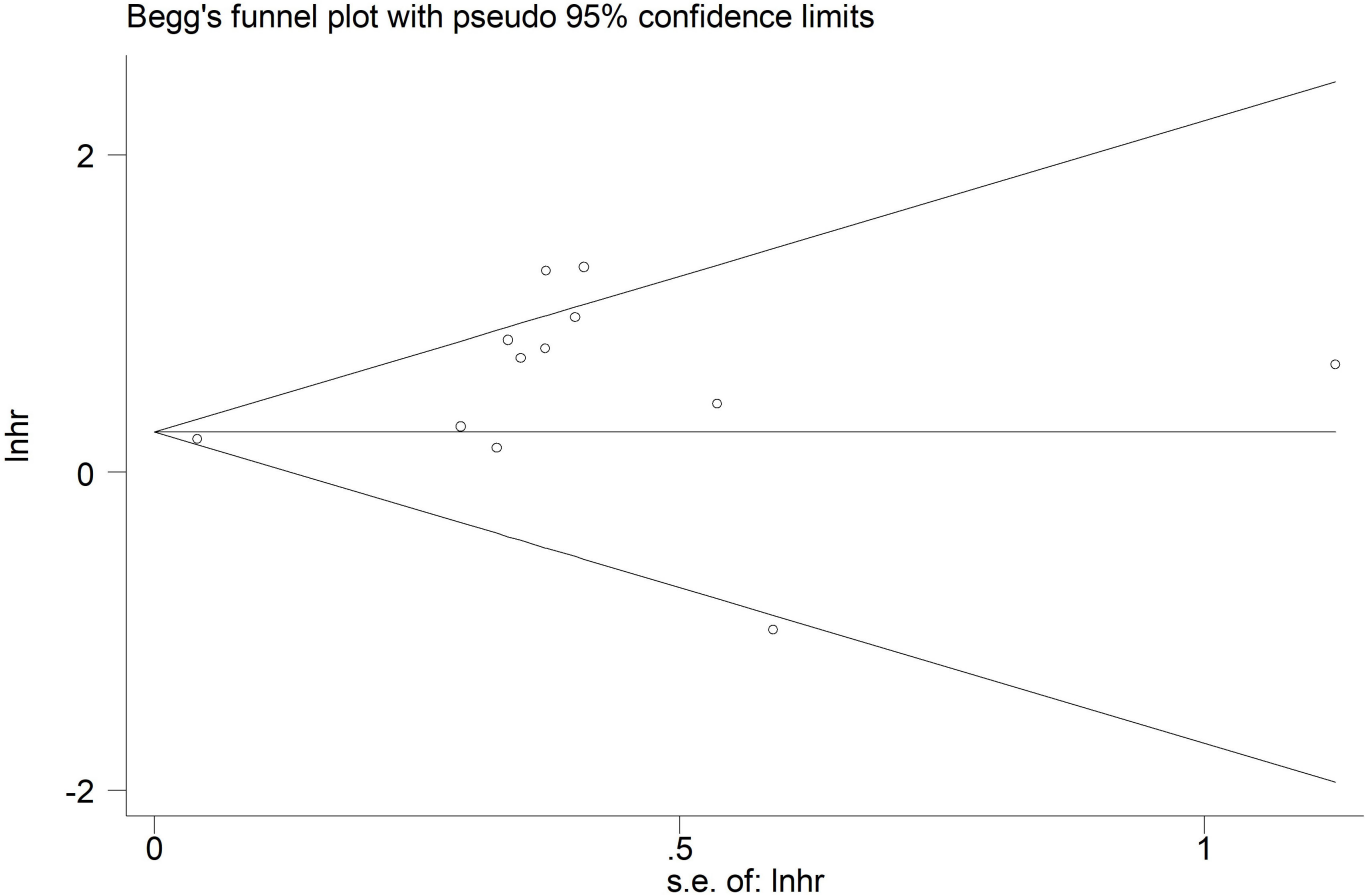
Begg’s funnel plots for the association of preoperative controlling nutritional status score with overall survival among surgical patients with esophageal cancer.

Based on Begg’s funnel plot (**Figure 6**) and Egger’s test (*p*=0.054), no statistically significant publication bias was detected. However, as the *p*-value was close to the conventional threshold of 0.05, a potential publication bias cannot be entirely ruled out.

## Discussion

According to our results, preoperative CONUT score may serve as a useful prognostic indicator in surgical esophageal cancer. Patients with an elevated CONUT score are exposed to significantly higher risk of worse clinical outcomes including the increased risk of postoperative complications and decreased long-term survival. However, owing to the limitations in included studies and this meta-analysis, more studies are still needed to further verify the above findings.

CONUT score is formulated according to the serum albumin level, cholesterol level, and total lymphocyte level. Cholesterol levels and lymphocyte counts in patients with esophageal cancer are closely related to their critical roles in the immune system and nutritional metabolism. Cholesterol is an essential component of cell membranes and participates in various physiological functions, such as cell signaling and hormone synthesis (35). Low cholesterol levels often indicate poor nutritional status, particularly in patients with cancer, where increased metabolic demands from the tumor and activation of inflammatory responses can reduce cholesterol synthesis (36). Moreover, low cholesterol levels are associated with impaired immune function, as cholesterol plays a crucial role in immune cell function (37). Studies have shown that patients with low cholesterol levels are more susceptible to infections, and tumor growth and metastasis may also be affected, leading to poorer prognosis (38). Lymphocytes are an important type of immune cell responsible for recognizing and eliminating abnormal cells, such as tumor cells, and foreign pathogens (39). Patients with cancer, particularly those with esophageal cancer, often face immune suppression, especially due to long-term disease progression and treatments like chemotherapy, which can result in reduced lymphocyte counts (40, 41). Low lymphocyte counts typically indicate a weakened immune system, unable to effectively identify and attack tumor cells, thereby affecting the anti-tumor immune response and increasing the risk of tumor recurrence or metastasis (40, 41).

In this context, the CONUT score may be viewed not merely as a simple nutritional index, but as a pragmatic surrogate that bridges systemic nutritional-immune status with underlying molecular and metabolic alterations in esophageal cancer. Its predictive value may therefore reflect, in part, the combined effects of nutritional deficiency, immune suppression, and tumor-metabolic rewiring. Future studies are warranted to validate its role in conjunction with more specific molecular biomarkers and to explore whether interventions to improve nutrition and immune function can favorably modulate the risk profiles indicated by CONUT.

Actually, there are two meta-analyses investigating the prognostic value of CONUT score in patients with esophageal cancer. Takagi et al. included five studies with only 952 patients and indicated that CONUT score was associated with OS (HR = 2.51, *p*<0.001), CSS (HR = 2.60, *p*<0.001), and DFS (HR = 2.08, *p*<0.001) among surgical patients with esophageal cancer (42). In another meta-analysis by Lv et al., 11 studies were included and similar associations between the pretreatment CONUT and survival were revealed (43). However, they did not focus on resected patients with esophageal cancer (43). In our meta-analysis, a total of 18 studies focusing on survival of patients with esophageal cancer were involved. Moreover, the prognostic role of CONUT score for short-term clinical outcomes presenting as postoperative complications was also systematically identified. In summary, our study provides stronger evidence for the application of CONUT score in the prognostic evaluation of patients with esophageal cancer who have undergone surgical treatment.

Although the pooled analysis demonstrated a significant association between elevated preoperative CONUT score and poorer OS, a moderate level of heterogeneity was observed (*I*² = 63.3%). This heterogeneity may be attributed to variations in patient characteristics and treatment strategies across the included studies. In particular, the proportion of patients receiving neoadjuvant therapy and the predominant pathological subtype differed among studies, both of which can substantially influence postoperative nutritional status and long-term outcomes. Subgroup analyses partly supported this explanation, as the association between CONUT score and OS remained significant in most subgroups, although it did not reach statistical significance in patients without neoadjuvant therapy. Moreover, differences in CONUT cutoff values, tumor stage distribution, and perioperative management practices could have contributed to the residual heterogeneity. These factors should be considered when interpreting the pooled results, and future studies with more standardized designs are warranted to further clarify these relationships.

Furthermore, the CONUT score provides a comprehensive reflection of a patient’s preoperative nutritional and immune status by simultaneously incorporating serum albumin, total cholesterol, and lymphocyte count. Compared with other commonly used indices such as the Prognostic Nutritional Index (PNI), Geriatric Nutritional Risk Index (GNRI), and neutrophil-to-lymphocyte ratio (NLR), CONUT has several clinical advantages. PNI and GNRI mainly evaluate protein reserves and body composition, while NLR emphasizes systemic inflammation. In contrast, CONUT integrates both nutritional and immunological dimensions, offering a more balanced assessment of the host’s metabolic and inflammatory state. Previous studies have suggested that CONUT exhibits superior prognostic performance for postoperative complications and long-term survival in gastrointestinal malignancies, including esophageal cancer, and can be easily obtained from routine laboratory data, making it a practical preoperative screening tool for risk stratification and perioperative optimization (23, 24, 44).

There are some limitations in this meta-analysis. First, most studies are from China or Japan, which might cause some bias. Second, it is hard to conduct more subgroup analyses based on other parameters such as the comparison method of CONUT score and age. Further research should investigate the influence of these parameters on the prognostic role of CONUT score in esophageal cancer. Third, since all included studies were retrospective in nature, potential confounding factors could not be fully eliminated. Therefore, the findings should be interpreted with caution, and prospective multicenter studies are warranted to validate the prognostic value of CONUT in esophageal cancer. Fourth, the language restriction may have introduced a potential language bias.

## Conclusion

Preoperative CONUT score might serve as a prognostic indicator in surgical esophageal cancer, and patients with an elevated CONUT score are more likely to experience worse long-term and short-term clinical outcomes. However, more studies are needed to further verify our conclusion.

## Data Availability

The original contributions presented in the study are included in the article/**Supplementary Material**. Further inquiries can be directed to the corresponding author.
