# Supplementary material for: Association of preoperative controlling nutritional status score with clinical outcomes among surgical patients with esophageal cancer: a meta-analysis
Source: Front Oncol. 2025 Nov 11;15:1694236. doi: 10.3389/fonc.2025.1694236 (PMC12643846; doi:10.3389/fonc.2025.1694236)
Supplement: Supplementary file 1 [file DataSheet1.docx]

Supplementary figure 1A. Subgroup analysis by the neoadjuvant therapy for the association of preoperative controlling nutritional status score with overall survival among surgical esophageal cancer patients.


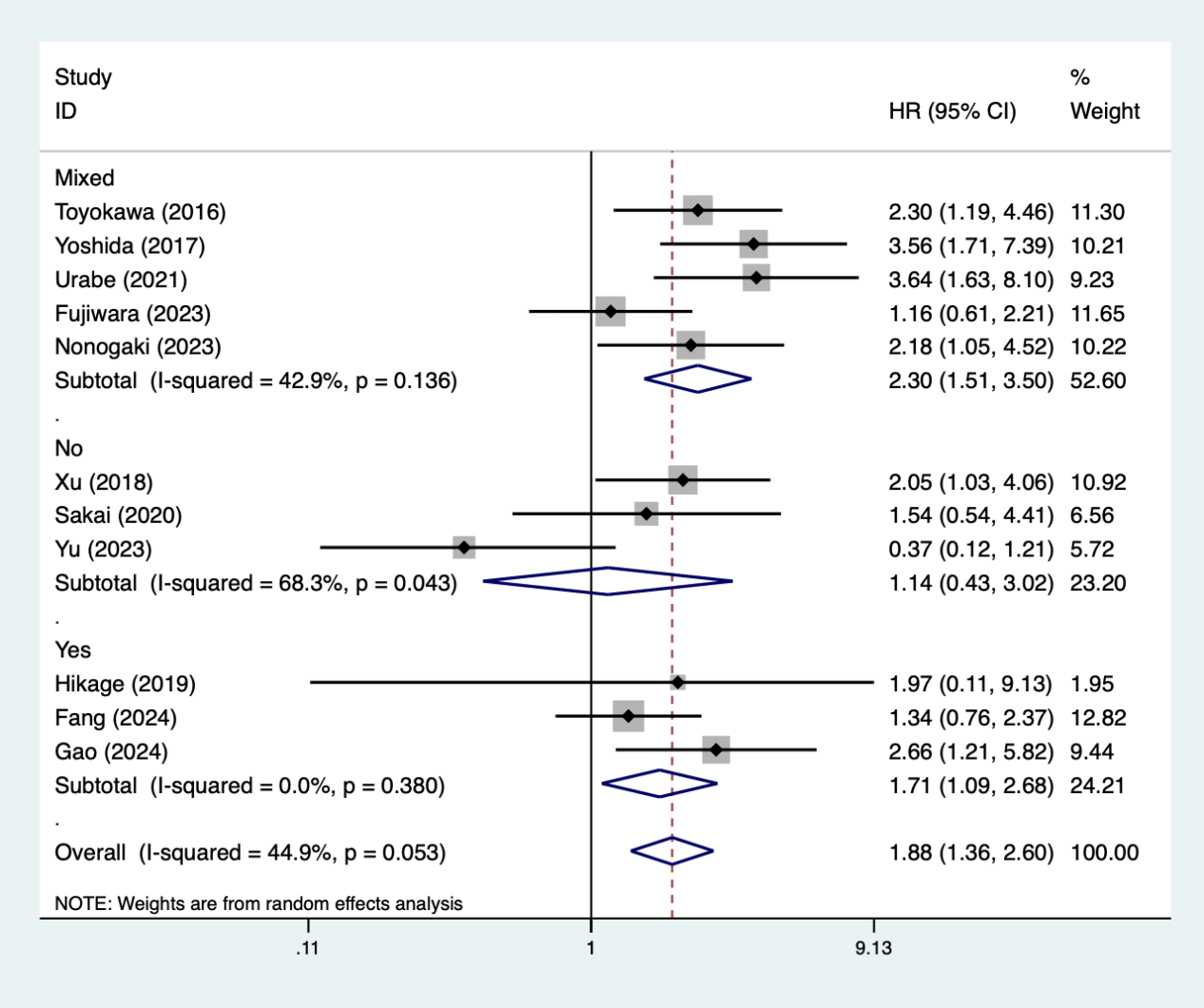


Supplementary figure 1B. Subgroup analysis by the pathological type for the association of preoperative controlling nutritional status score with overall survival among surgical esophageal cancer patients.


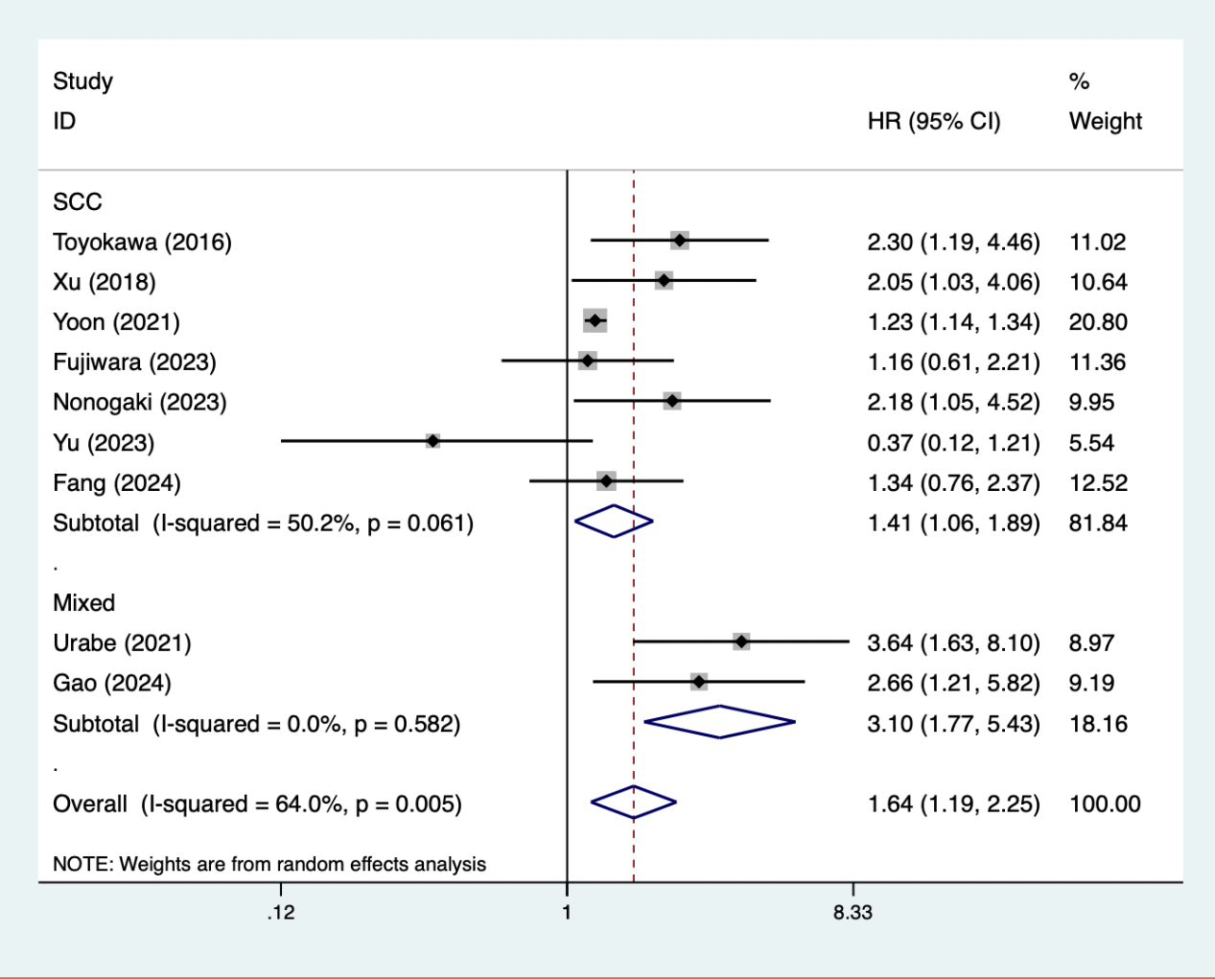


Supplementary figure 1C. Subgroup analysis by the neoadjuvant therapy for the association of preoperative controlling nutritional status score with disease-free survival among surgical esophageal cancer patients.


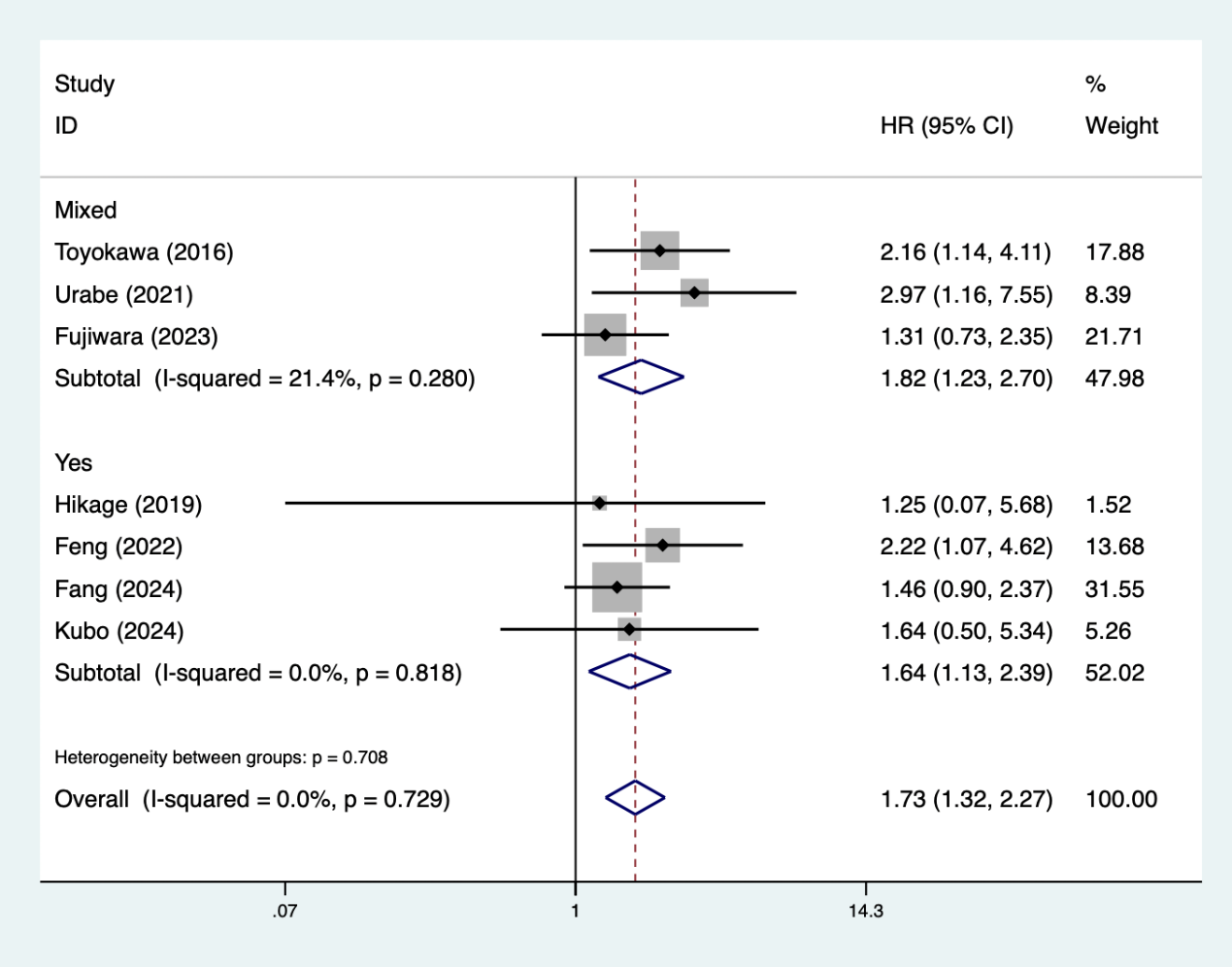


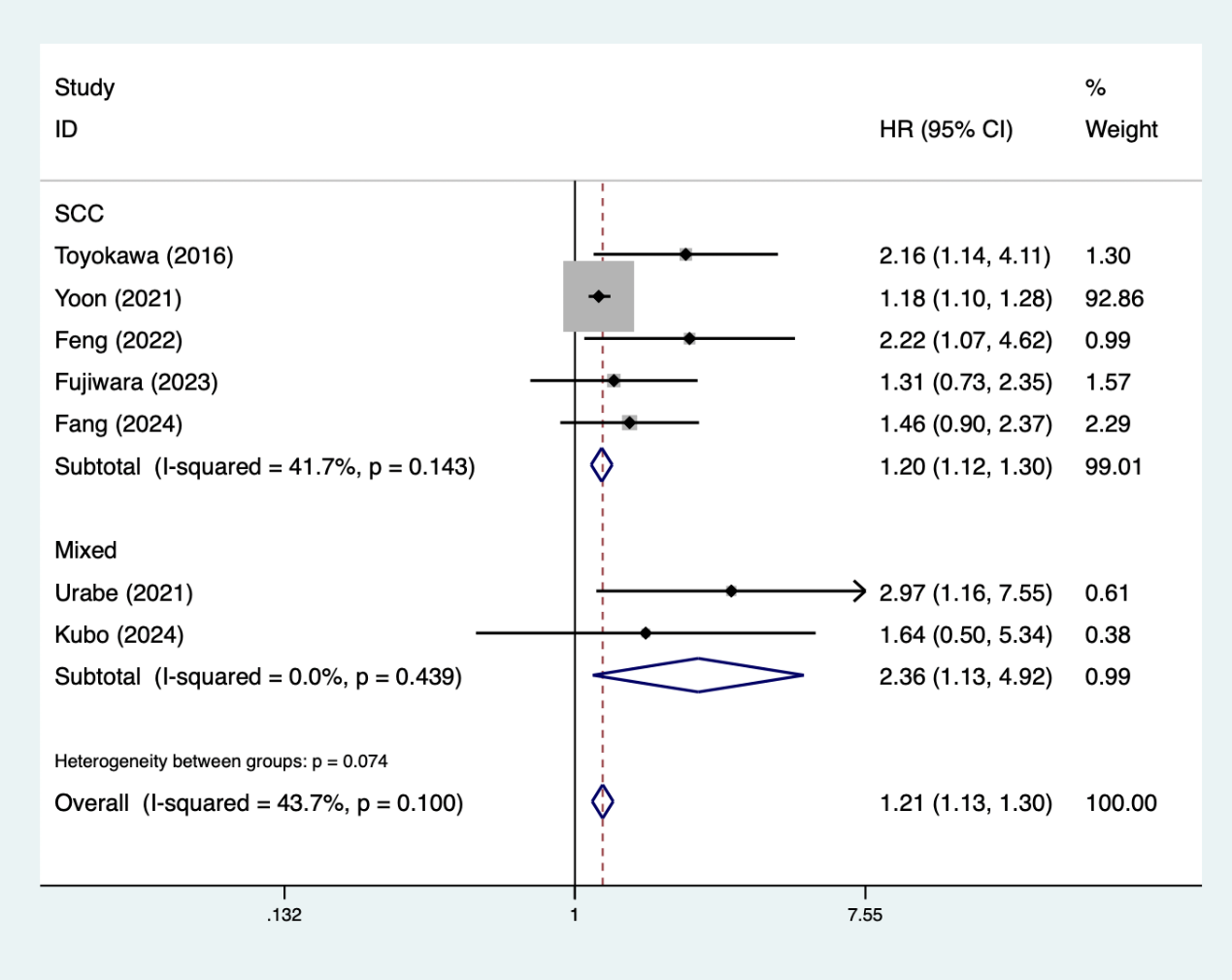
Supplementary figure 1D. Subgroup analysis by the pathological type for the association of preoperative controlling nutritional status score with disease-free survival among surgical esophageal cancer patients.
