# Supplementary material for: Association of preoperative controlling nutritional status score with clinical outcomes among surgical patients with esophageal cancer: a meta-analysis
Source: Front Oncol. 2025 Nov 11;15:1694236. doi: 10.3389/fonc.2025.1694236 (PMC12643846; doi:10.3389/fonc.2025.1694236)

Supplementary figure 2A. Association of preoperative controlling nutritional status score with postoperative pneumonia among surgical esophageal cancer patients.


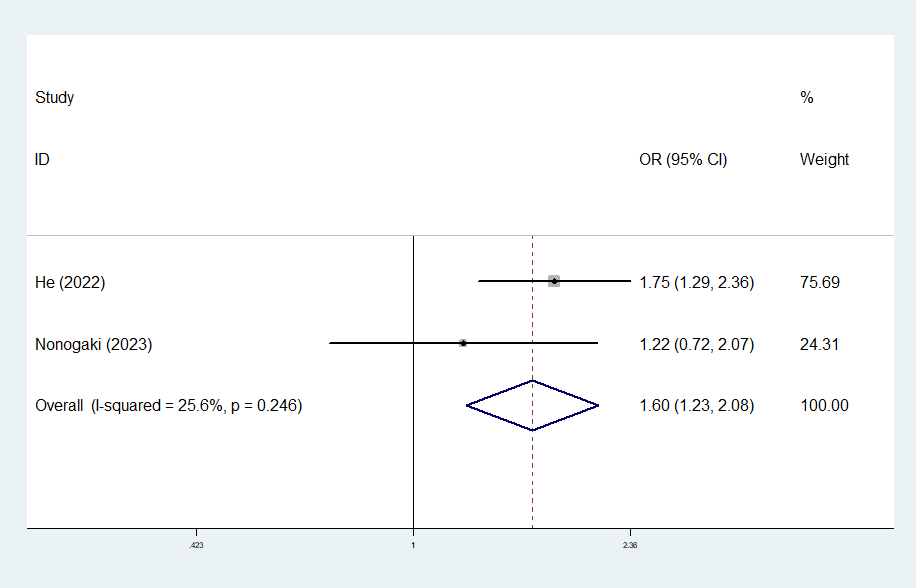


Supplementary figure 2B. Association of preoperative controlling nutritional status score with postoperative respiratory complication among surgical esophageal cancer patients.


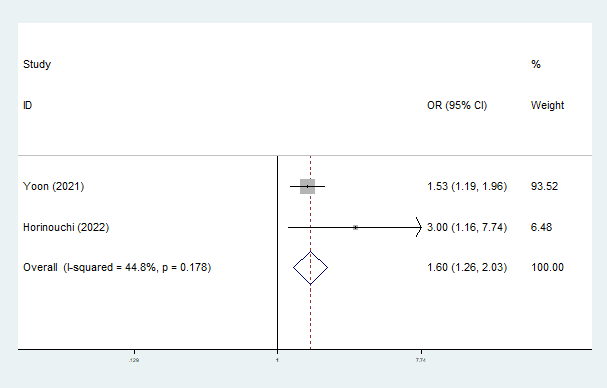

Supplement: Supplementary file 2 [file DataSheet2.docx]
