# Supplementary figures and images for: Association of preoperative controlling nutritional status score with clinical outcomes among surgical patients with esophageal cancer: a meta-analysis
Source: Front Oncol. 2025 Nov 11;15:1694236. doi: 10.3389/fonc.2025.1694236 (PMC12643846; doi:10.3389/fonc.2025.1694236)

Supplementary file1. The detailed search strategy in the PubMed.


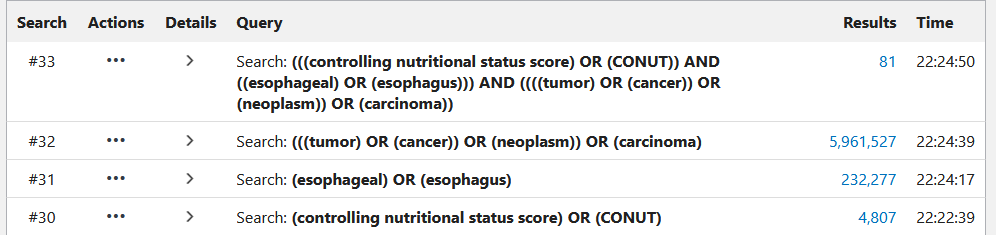

Supplement: Supplementary file 3 [file DataSheet3.docx]
