# Supplementary material for: Association of preoperative controlling nutritional status score with clinical outcomes among surgical patients with esophageal cancer: a meta-analysis
Source: Front Oncol. 2025 Nov 11;15:1694236. doi: 10.3389/fonc.2025.1694236 (PMC12643846; doi:10.3389/fonc.2025.1694236)
Supplement: Supplementary file 4 [file DataSheet4.docx]

Supplementary table 1. Detailed results of NOS score.

| Author | Selection | | | | Comparability | Outcome measurement | | | Score |
| --- | --- | --- | --- | --- | --- | --- | --- | --- | --- |
|  | Representativeness of the exposed cohort | Selection of the non-exposed cohort | Ascertainment of exposure | Outcome of interest | Comparability of cohorts | Assessment of outcome | Time of follow-up | Adequacy of follow-up |  |
| Toyokawa [18] | 1 | 1 | 1 | 1 | 0 | 1 | 1 | 1 | 7 |
| Yoshida [19] | 1 | 1 | 1 | 1 | 0 | 1 | 1 | 1 | 7 |
| Hirahara [20] | 1 | 1 | 1 | 1 | 0 | 1 | 1 | 1 | 7 |
| Xu [21] | 1 | 1 | 1 | 1 | 1 | 1 | 1 | 1 | 8 |
| Hikage [22] | 1 | 1 | 1 | 1 | 0 | 1 | 1 | 1 | 7 |
| Sakai [23] | 0 | 1 | 1 | 1 | 0 | 1 | 1 | 1 | 6 |
| Yoon [24] | 1 | 1 | 1 | 1 | 1 | 1 | 1 | 1 | 8 |
| Urabe [25] | 1 | 1 | 1 | 1 | 0 | 1 | 1 | 1 | 7 |
| Wang [26] | 1 | 1 | 1 | 1 | 0 | 1 | 1 | 1 | 7 |
| Feng [27] | 1 | 1 | 1 | 1 | 0 | 1 | 1 | 1 | 7 |
| He [28] | 1 | 1 | 1 | 1 | 0 | 1 | 1 | 1 | 7 |
| Horinouchi [29] | 1 | 1 | 1 | 1 | 1 | 1 | 1 | 1 | 8 |
| Fujiwara [30] | 1 | 1 | 1 | 1 | 0 | 1 | 1 | 1 | 7 |
| Nonogaki [31] | 1 | 1 | 1 | 1 | 0 | 1 | 1 | 1 | 7 |
| Yu [32] | 0 | 1 | 1 | 1 | 0 | 1 | 1 | 1 | 6 |
| Fang [33] | 1 | 1 | 1 | 1 | 0 | 1 | 1 | 1 | 7 |
| Gao [34] | 1 | 1 | 1 | 1 | 0 | 1 | 1 | 1 | 7 |
| Kubo [35] | 1 | 1 | 1 | 1 | 0 | 1 | 1 | 1 | 7 |

NOS: Newcastle-Ottawa Scale.
